# Intradialytic resistance training: an effective and easy-to-execute strategy

**DOI:** 10.1590/2175-8239-JBN-2018-0134

**Published:** 2018-11-08

**Authors:** Antônio Paulo André de Castro, Sergio Ribeiro Barbosa, Henrique Novais Mansur, Danielle Guedes Andrade Ezequiel, Mônica Barros Costa, Rogério Baumgratz de Paula

**Affiliations:** 1 Universidade Federal de Juiz de Fora Faculdade de Medicina Programa de Pós-graduação em Saúde Juiz de ForaMG Brasil Universidade Federal de Juiz de Fora, Programa de Pós-graduação em Saúde da Faculdade de Medicina, Juiz de Fora, MG, Brasil.; 2 Centro de Ensino Superior de Valença ValençaRJ Brasil Centro de Ensino Superior de Valença, Valença, RJ, Brasil.; 3 Faculdade do Sudeste Mineiro Juiz de ForaMG Brasil Faculdade do Sudeste Mineiro, Juiz de Fora, MG, Brasil.; 4 Faculdade de São Lourenço São LourençoMG Brasil Faculdade de São Lourenço, São Lourenço, MG, Brasil.; 5 Instituto Federal do Sudeste de Minas Gerais Rio PombaMG Brasil Instituto Federal do Sudeste de Minas Gerais, Rio Pomba, MG, Brasil.; 6 Universidade Federal de Juiz de Fora Juiz de ForaMG Brasil Universidade Federal de Juiz de Fora, Juiz de Fora, MG, Brasil.

**Keywords:** Resistance Training, Renal Insufficiency, Renal Dialysis, Quality of Life, Muscle Strength

## Abstract

Chronic kidney disease (CKD) alters the morphology and function of skeletal muscles, thereby decreasing patient physical capacity (PC) and quality of life (QoL). Intradialytic resistance training (IRT) is a pragmatic tool used to attenuate these complications. However, IRT has not been strongly adopted in nephrology care centers. This study aimed to assess the efficacy and safety of a low-cost, easy-to-use IRT protocol.

**Methods:**

The study enrolled 43 patients (52.8 ± 13.85 years) on HD for five to 300 months followed from April 2014 to July 2017. The efficacy of IRT was assessed based on PC - derived from muscle strength (MS) and preferred walking speed (PWS) - and QoL. The occurrence of adverse events was used as a measure of safety. The IRT protocol consisted of exercises of moderate to high intensity for the main muscle groups performed three times a week.

**Results:**

The mean follow-up time was 9.3 ± 3.24 months, for a total of 4,374 sessions of IRT. Compliance to the protocol was 96.5 ± 2.90%, and patients presented significant improvements in MS (from 27.3 ± 11.58 Kgf to 34.8 ± 10.77 Kgf) and PWS (from 0.99 ± 0.29 m/s to 1.26 ± 0.22 m/s). Physical and emotional components of QoL also increased significantly.

**Conclusion:**

IRT led to significant increases in PC and higher scores in all domains of QoL. Important adverse events were not observed during intradialytic resistance training.

## Introduction

Individuals with chronic kidney disease (CKD) suffer from alterations in the morphology and function of skeletal muscles, which translate into weakness and gradual decreases in physical capacity (PC) and quality of life (QoL).^1^^,^^2^ In recent years, some of the guidelines published in nephrology - the K/DOQI in particular - have advocated the introduction of exercise training as a measure to attenuate complications and decrease the occurrence of adverse outcomes such as loss of autonomy, increased risk of falls, endocrine and metabolic disorders, and higher hospitalization rates mainly for cardiovascular events.^3^^-^6 Many authors have written about the benefits of exercise training - and specifically aerobic exercises - at improving PC and QoL in patients with CKD.^7^^,^^8^ Studies carried out by our group revealed that aerobic exercises were safe and produced increases in VO_2_ max and better PC and QoL.^9^^,^^10^ Although beneficial, intradialytic resistance training (IRT) still faces a few obstacles in attaining greater levels of acceptance in clinical practice on account of the high costs associated with procuring and maintaining exercise equipment and the need to modify the room in which equipment is installed. Additionally, patients with CKD are often unable to bear the volume and intensity required for effective aerobic training for reasons such as low levels of cardiorespiratory fitness; lower limb bone, muscle, and joint limitations; and femoral dialysis catheters. Therefore, few dialysis centers in Brazil offer IRT.

Few studies have looked into moderate to high intensity IRT, a method with great potential for improving muscle strength (MS) and PC.^11^^,^^12^ Headley et al.^13^ studied patients on a 12-week low-intensity IRT protocol and observed a 13% increase in the strength of knee extensor muscles. Kirkman et al.^14^ corroborated these findings as they analyzed a group of patients on a 12-week IRT program of moderate-to-high intensity using sophisticated training equipment. The authors reported a 60% increase in the strength of a specific muscle group in 23 patients. Preliminary data from our service show that MS, preferred walking speed (PWS), and QoL improved after three months of IRT using low cost equipment (ankle weights and dumbbells). Although results are promising, questions over the safety of IRT and the lack of knowledge from health care workers still seem to pose barriers to a wider adoption of exercise training programs in nephrology centers.^15^^,^^16^

This study aimed to assess the efficacy and safety of a moderate-to-high intensity, easy-to-execute and affordable intradialytic resistance training protocol.

## Methods

This prospective controlled study with supervised intervention was carried out from April 2014 to July 2017. The Research Ethics Committee of the Hospital Universitário da Universidade Federal de Juiz de Fora approved the study (opinion 375.003).

### Patients

Adult patients of both sexes on hemodialysis for at least three months were included in the study. Information concerning the assessment and training protocols were shared with the patients, who voluntarily signed an informed consent term approved by the Research Ethics Committee of the Hospital Universitário da Universidade Federal de Juiz de Fora (CAAE: 20145613.4.0000.5133; no. 375.003).

**Figure 1:**
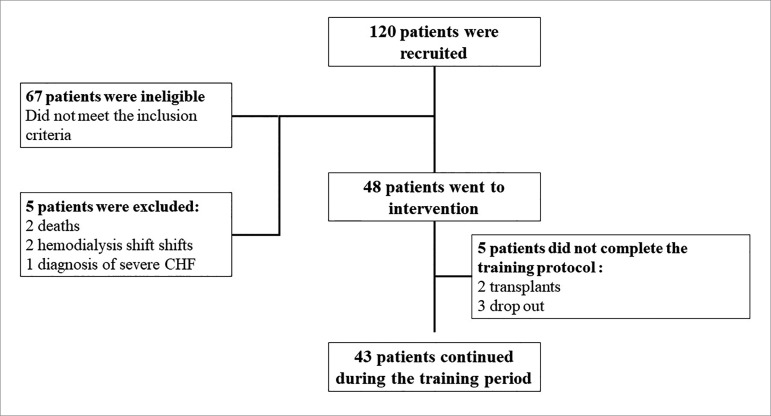
Patient enrollment workflow

The following exclusion criteria were applied: hypoalbuminemia (serum albumin < 3 g/dL), fasting glucose > 300 mg/dL, unstable angina, heart arrhythmia, decompensated heart failure, uncontrolled hypertension defined as systolic blood pressure (SBP) ≥ 200 mmHg and/or diastolic blood pressure (DBP) ≥ 120 mmHg, uremic pericarditis, severe lung disease, acute systemic infection, severe renal osteodystrophy, and musculoskeletal disorders preventing the patients from performing the exercises. Figure 1 shows the patient enrollment workflow.

Before initiating the physical training program, all patients underwent cardiologic, anthropometric, and PC assessment. Cardiologic assessment included an interview, physical examination, and exercise tests to detect possible physical effort-induced cardiovascular disorders. In anthropometric evaluation, the body mass index (BMI) was calculated based on the dry weight. PC was analyzed based on the hand grip strength test, the 30-second chair stand test, and the 15-foot walk test to find the PWS. All tests were carried out on days between dialysis sessions. The Medical Outcomes Study 36-Item Short Form Health Survey (SF-36) was applied as an interview to assess patient QoL. The SF-36 comprises 36 items in the following scales: physical functioning, role-physical, bodily pain, general health, vitality, social functioning, role-emotional, and mental health. For each of the scales patients can score from 0 (worst QoL) to 100 (best QoL). Demographic data, clinical, and workup parameters were gathered from the patients' charts and HD session records.

The effect of moderate-to-high intensity IRT on MS, PC, and QoL was investigated as a primary endpoint. The secondary endpoint was the impact of IRT on the quality of dialysis analyzed via the Kt/V. This parameter was calculated based on the ratio between the product of the dialyzer clearance of urea (K) and dialysis time (t) over the volume of distribution of urea clearance (V).

### Experimental protocol

The patients had resting blood pressure and heart rate measured before the start of the IRT sessions. Diabetic individuals also had their capillary blood glucose checked. With safety in mind, patients were allowed to start IRT sessions as long as the SBP ranged between 110 and 160 mmHg and/or DBP were between 50 and 100 mmHg and if their resting heart rate were between 50 and 100 bpm. The capillary blood glucose of diabetic patients had to be between 100 and 250 mg/dL.

**Figure 2:**
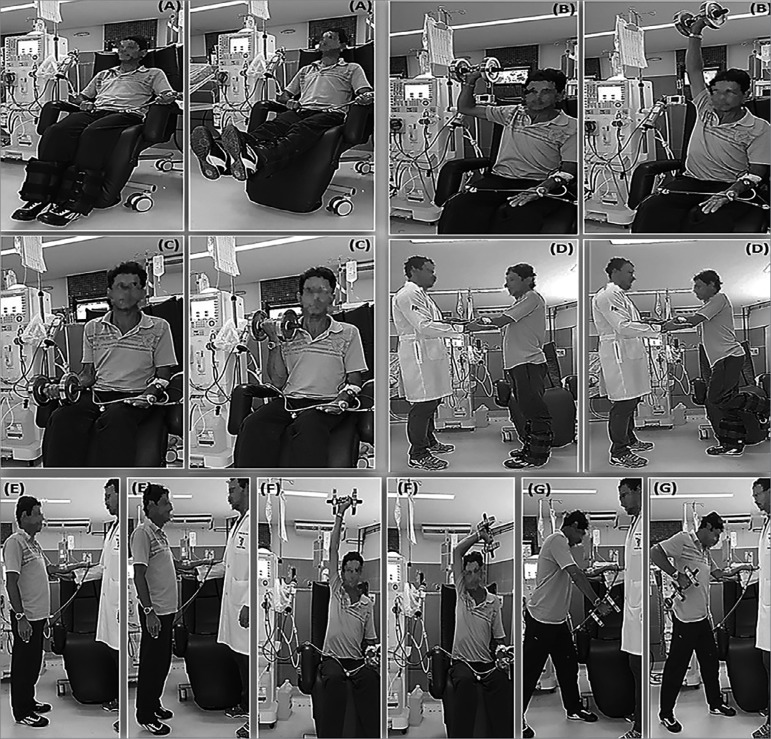
Initial proposal of a resistance training protocol for patients on hemodialysis. Notes: (a) bilateral knee extension with ankle weights; (b) unilateral shoulder abduction and elbow extension with dumbbells (shoulder development); (c) unilateral elbow flexion with dumbbells (biceps curl); (d) alternating knee flexion with ankle weights; (e) bilateral plantar flexion (free calf); (f) unilateral elbow extension with dumbbells (French press); (g) unilateral shoulder extension and elbow flexion with dumbbells (row).

**Figure 3:**
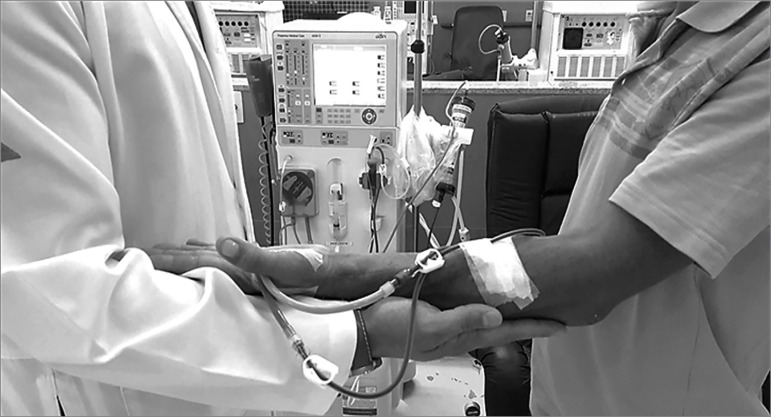
Technique used by a physical educator to support the arteriovenous fistula arm of a patient on hemodialysis during standing resistance training.

Physical education professionals supervised the patients throughout the IRT sessions three times a week. The training sessions were carried out during the first two hours of HD and lasted for approximately 50 minutes. The proposed IRT (Figure 2) consisted of exercises for the main muscle groups (dorsal muscles: unilateral standing row; pectoral muscles: flat bench press; deltoid muscles: seated shoulder press; quadriceps: knee extension; hamstrings: knee flexion; calf muscle: plantar flexion in an orthostatic position; brachial triceps: unilateral French press; brachial biceps: unilateral curl). In order to perform the exercises in an orthostatic position, the patients were aided by a physical educator to stabilize their arms with the arteriovenous fistula (Figure 3).

In the first week of the protocol (familiarization stage) the patients were requested to perform only one set of 10 to 15 repetitions for each of the exercises. In the second week they moved up to two sets of 10 to 15 repetitions. From the third week onwards they performed three sets of 10 to 15 repetitions. The Borg rating of perceived exertion (RPE) was used to determine and manage effort intensity in all stages of the protocol.^17^ Individuals were asked, in plain terms, to assess the training load considering central (e.g.: lung ventilation) and peripheral factors (muscles and joints). After assessing the level of effort, the patients were asked to assign a score to their perceived exertion on a scale ranging from 6 to 20, in which 6 meant less exertion and 20 the highest possible level of exertion. The enrolled patients performed exercises scored between 15 and 17, the equivalent of "strong" and "very strong" perceived effort. At the end of each set and exercise, the patients were asked about their RPE. If they scored outside the study range, the load was adjusted by about 5% to either increase or decrease it. In order to manage intensity, patients performing all three sets with 15 repetitions had the load readjusted by about 5% the following session. In all stages of the protocol patients were given 90 to 120 seconds to rest between sets and exercises. In order to avoid early muscle fatigue, the exercises were performed alternating between segments as per the guidelines set out by the American College of Sports Medicine.^18^

## Results

A total of 120 patients were initially selected, but 48 ended up being enrolled (40%) and 43 stayed until the end. The mean age of the included patients was 52.8 ± 13.85 years, as characteristically seen in populations on HD. Most were males, and time on HD ranged from five to 300 months. Demographic and clinical variables at the start of the protocol are described on Table 1.

**Table 1:** Demographic and clinical findings of the study population

Variable	Total (n = 43)
Sex [male: female; n (%)]	37:16 (70/30%)
Age [years; mean (SD)]	52.8 (13.85)
Time on hemodialysis [months; median (IQR)]	36 (17 - 105)
Kt/V [mean (SD)]	1.47 (0.50)
Body mass index [kg/m^2^; mean (SD)]	26.0 (7.40)
Etiology of CKD [n (%)]	
Hypertensive nephrosclerosis	29 (54.7%)
Glomerulonephritis	8 (15.1%)
Diabetic kidney disease	8 (15.1%)
Polycystic kidney disease	1 (1.9%)

The total time of intervention was 39 months, with a mean follow-up time of 9.3 ± 3.24 months and a total of 4,374 individual IRT sessions. Compliance to protocol was 96.5 ± 2.90%, and only 0.80% of the sessions were not carried out for reasons such as uncontrolled blood pressure, pain or mismatches with the HD schedule. No significant complications were observed during the IRT sessions. Only one case of a hematoma associated with the arteriovenous fistula was recorded due to patient neglect.

**Figure 4:**
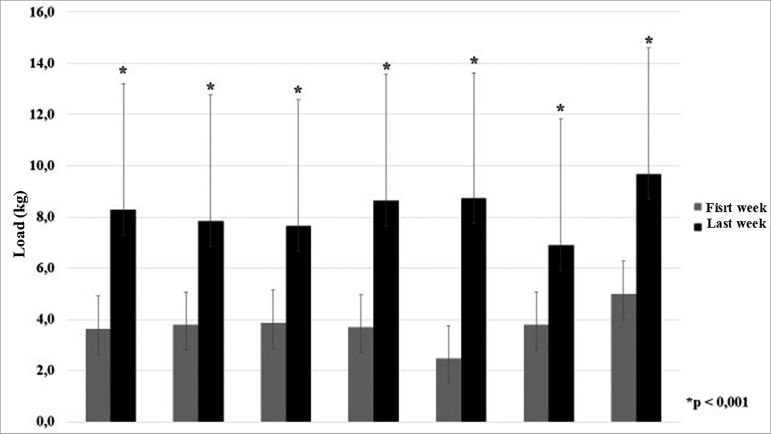
Gradual exercise load increases: initial vs. final load. Notes: 1: knee extension; 2: development; 3: elbow flexion; 4: knee flexion; 5: plantar flexion; 6: elbow extension; 7: unilateral row; * significant difference between initial and final load.

The load for all exercises was gradually increased throughout the weeks of training. The loads used in the first and last week of the program were statistically different (*p* < 0.001). Load progression was similar in all exercises and ranged from 180% to 440% of the initial load (Figure 4). Consequently, MS increased significantly from 27.3 ± 11.58 Kgf to 34.8 ± 10.77 Kgf (*p* = 0.004). In addition to increasing the training load, patients improved PC as a function of the PWS, which grew from an initial 0.99 ± 0.29 m/s to 1.26 ± 0.22 m/s (*p* = 0.0003).

**Figure 5:**
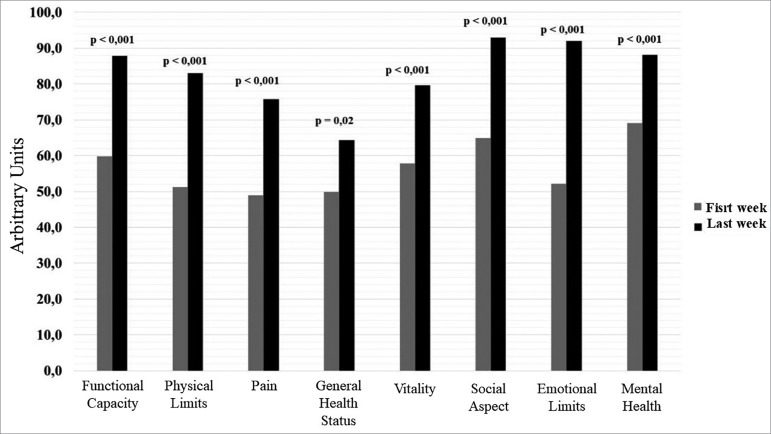
Comparison between initial and final scores in the assessed quality-of-life scales.

QoL also improved when the first and last weeks of training were compared, both in the domains associated with physical and emotional elements (Figure 5).

At the end of the follow-up period, a non-significant improvement was seen in the quality o dialysis assessed by Kt/V, which moved from 1.4 ± 0.50 to 1.6 ± 0.36.

## Discussion

This study presented data on the efficacy and safety of an individualized resistance training protocol supervised by physical educators offered to patients during hemodialysis sessions. The training model used in this study was based on the use of low cost materials and produced increases in MS, PC, and QoL improvements. The protocol was well accepted and tolerated by the patients, and no significant adverse events were recorded, which characterized it as a safe, affordable, and easy-to-execute method.

Although technological advancements related to improved quality of dialysis and comorbidity management have been implemented in recent years, patients with CKD still present with lower levels of MS and PC when compared to the population in general. Several studies have shown that exercise training may positively impact these variables.^7^^,^^8^ Although most studies were conducted using aerobic exercises alone or combined with low intensity resistance training, recent evidence indicates that the benefits of moderate and high intensity resistance training may be superior at increasing MS.

An increase of 45% in MS was seen in our study, as similarly reported by Molsted et al.^19^ and Kirkman et al.,^14^ who reported respective increases of 46% and 60% after high intensity training using sophisticated equipment. Chen et al.^20^ and Chan et al.^21^ did not report significant increases in MS, possibly because in their studies the patients were offered low to moderate intensity exercise training.

In addition to serving as an indicator of global health status, PWS is a known marker of PC and a predictor of risk of death for all causes and cardiovascular disease. Individuals with CKD have lower PWS than the general population.^22^ Kutner et al.^23^ studied 752 patients and noticed that lower PWS was associated with greater risk of death. In our study, initial PWS was low and similar to the values reported by other authors.^24^^-^26 PWS increased significant after exercise training, as also reported by Headley et al.,^13^ Bennett et al.,^27^ and Anding et al.^28^ Johansen et al.^29^ and Corrêa et al.^30^ did not report significant increases in PWS, possibly because in their studies patients performed low intensity training.

In recent years, the QoL of individuals with CKD has captured the attention of health care workers. CKD of all stages significantly compromises all QoL domains. Despite the advancements achieved in the treatment of CKD, improving the QoL of patients with this condition is still a challenge in clinical practice. Strategies such as nutritional plans, psychotherapy, and compliance improvements are examples of actions devised to recuperate patient QoL.^4^ Various studies have also shown that the lower level of QoL observed in individuals with CKD when compared to the general population is invariably associated with increased morbimortality.^31^^,^^32^ Other factors that may compromise QoL include decreased MS and PC.^33^ The QoL of the patients enrolled in our study increased significantly in physical and emotional domains after the introduction of exercise training. The improvements seen in our study were greater than the improvements reported by Johansen et al.,^29^ Bennett et al.,^27^ Corrêa et al.,^30^ and Rosa et al.,^34^ partly on account of continuous supervision by a physical educator and training intensity and length.

With compliance rates close to 95% and interruptions in only 0.8% of the sessions, the results reported in this study were similar to the findings reported by Kirkman et al.^14^ and were notably superior when compared to the results described by Headley et al.,^13^ DePaul et al.,^25^ Nindl et al.,^35^ Chen et al.,^20^ and 36 Martin-Alemañy et al.^36^ Our results and the improved levels of MS, PC, and QoL may be attributed to the exercise protocol implemented during HD sessions, the progressive adjustment of exercise loads in accordance with the guidelines of the American College of Sports Medicine,^18^ and supervision by a physical educator.

Exercise training has been tried experimentally with patients with CKD for more than three decades with proven benefits. Nevertheless, the practice is still seen with reserve at nephrology centers. It has been speculated that factors associated with the disease itself - including anemia, fatigue, and exertion intolerance - in addition to fear of clinical complications, unawareness of the benefits of exercise training, lack of training on the delivery of IRT, and low patient motivation act as barriers to the implementation of exercise training programs in kidney centers.^37^^-^39 The concern of health workers with clinical complications happening during physical exercise is valid. However, a meta-analysis published by Cheema et al.^40^ found that the risk of adverse events occurring during exercise training is low. Accordingly, our study had only one case of a patient with a hematoma in the arm of the arteriovenous fistula after an exercise session, an adverse event not directly related to IRT.

On account of its physiological and methodological characteristics, IRT allows patients to pause between exercise sets and use different muscle groups, thus minimizing fatigue and exercise intolerance. Besides, HD sessions become less tedious and some patients find additional motivation and relief in exercises while undergoing hemodialysis. The IRT protocol described in this paper requires low cost materials (dumbbells and ankle weights) and no changes to HD rooms, as is the case of exercise programs delivered on cycle ergometers.

A recent meta-analysis showed that the combination of resistance and aerobic training produced superior effects on PC and QoL when compared to each training mode done separately.^41^ Further clinical trials comparing the effectiveness of aerobic and resistance training at different levels of intensity, including a scenario in which patients do exercises at home, may contribute to the optimization of exercise training for patients with CKD on renal replacement therapy.

## Conclusion

Supervised IRT significantly increased MS and PC and improved the overall QoL of patients with CKD. The easy-to-execute and affordable protocol described in this paper was effective and did not correlate with significant adverse events, which opens the doors to the implementation of this mode of conservative management for individuals with CKD as recommended in recent nephrology guidelines.
